# Consumer Attitudes and Purchase Intentions in Relation to Animal Welfare-Friendly Products: Evidence from Taiwan

**DOI:** 10.3390/nu14214571

**Published:** 2022-10-31

**Authors:** Min-Yen Chang, Han-Shen Chen

**Affiliations:** 1 Department of Accounting, Jiaxing University, Jiaxing 314001, China; 2 Department of Health Industry Technology Management, Chung Shan Medical University, Taichung 40201, Taiwan; 3 Department of Medical Management, Chung Shan Medical University Hospital, Taichung 40201, Taiwan

**Keywords:** sustainable development goals (SDG_S_), food safety, food labelling, moral affection, health consciousness

## Abstract

Animal welfare, environmental sustainability, and food safety have become topics of international concern. With the rise of friendly rearing and green consumption consciousness, consumers can use animal welfare certification labels as references to make purchase decisions. This study adopts the theory of planned behavior (TPB) as its core and incorporates variables, such as moral affection, health consciousness, and trust in certification, to discuss the thoughts of Taiwanese consumers on buying animal welfare-friendly products and the factors that affect their purchase decisions. This study will be conducive in clarifying the consumption behavioral pattern of animal welfare-friendly products, which previous literature has mentioned but not tested, thereby filling this literature gap. This study collects 653 valid questionnaires and uses the partial least square-structural equation modeling to analyze the correlations between various variables. The research findings indicate the following. (1) Consumers’ attitudes, subjective norms, and perceived behavioral control have significant and positive influences on the behavioral intention of purchasing fresh milk with animal welfare labels. (2) Moral affection positively influences customers’ behavioral intention toward fresh milk with an animal welfare label through attitudes, subjective norms, and perceived behavioral control. (3) Trust in certifications will enhance moral cognition and positive attitudes toward fresh milk with animal welfare labels. According to the research findings of this study, we recommend that businesses strengthen the promotion of dairy products in line with friendly rearing, environmental sustainability, and other ethical consumption concepts to generate market segregation elements.

## 1. Introduction

According to a report by the United Nations Environment Programme UNEP) (2021) [1], reducing methane emissions is the fastest, most effective, and economic measure to slow down the greenhouse effect. Doing so will help control the rise in the global temperature to within 1.5 degrees Celsius. More than half of the anthropogenic methane emissions worldwide originate from the three sectors, namely, fossil fuel, waste, and agriculture. Of these, in the agricultural sector, livestock manure and the enteric fermentation of ruminants account for 32% of anthropogenic emissions worldwide. Health and environmental burdens caused by the excessive consumption of livestock and poultry products have become prevalent in developed and developing countries globally. Moreover, large-scale and extensive industrialized rearing seriously affects animal welfare, health, and food safety, whereas livestock waste exacerbates global warming. Therefore, reducing the quantity of livestock rearing and encouraging farmers to shift to friendly rearing can reduce animal enteric fermentation and manure and urine processing, thus slowing down global warming. In 2019, the European Union (EU) passed the European Green Deal, which aims to facilitate the comprehensive transformation of the EU to achieve carbon neutrality by 2050, reduce greenhouse gas emissions, and curb the continuous depletion of biology and environmental resources in Europe and around the world, keeping in line with the sustainable development goals.

Animal welfare not only involves ethical and humane considerations but also concerns food safety, disease prevention and control, and the sustainable development of husbandry. The most fundamental idea of Farm Animal Welfare (FAW) is that it hopes that animals can enjoy five freedoms, namely, “freedom from hunger and malnutrition”, “freedom from fear or distress”, “freedom to express normal behavior”, “freedom from pain and disease”, and “freedom from enduring discomfort due to the environment” [2]. Since 2014, the Council of Agriculture (COA), Executive Yuan in Taiwan also successively developed definitions and guidelines of the animal-friendly production system for eggs, pigs, cow milk, and other livestock and poultry products. In 2019, COA released the White Paper on Animal Welfare, hoping to turn Taiwan’s husbandry in a friendly development direction. To further develop a fair, healthy, and environmentally friendly food system, the EU passed the Farm to Fork Strategy on 20 May 2020, expecting to transform the European food system into a sustainable one and reverse the loss of biodiversity and detrimental environmental trends around the world, simultaneously ensuring food safety and affordability. The action plan comprises the following: evaluate and revise animal welfare laws and regulations (e.g., ban on the domestic cage rearing of economic animals from 2027 onward) to promote forms of economic animal welfare and healthy and sustainable food consumption, giving consumers full access to healthy dietary information, thereby choosing food that is friendly to the environment and animals [3].

In response to consumers’ demand and attention, animal welfare-friendly products have entered the fast food chain restaurant system. For example, in the United Kingdom, Burger King publishes its animal welfare policy on its website, stating that all of the milk, meat, and egg products that it uses come from farms conforming to EU regulations on animal welfare. In the United Kingdom, McDonald’s has also stated in the food source column on its website that all of the eggs that it uses come from farms conforming to the animal welfare standards of the Royal Society for the Prevention of Cruelty to Animals (RSPCA). In Taiwan, Carrefour announced in 2018 that it would set up a dedicated area for animal welfare-friendly eggs in their supermarkets, increasing the visibility of animal welfare-friendly products. An increasing number of animal welfare-friendly eggs have appeared in grocery stores and superstores, indicating that animal welfare has gradually transformed into a value added product, forming an element for market segregation.

Currently, the following “animal welfare certification” labels with an international reputation and credibility are used: RSPCA Assured of the United Kingdom; Beter Leven of the Netherlands; Animal Welfare Approved, promoted by A Green World of the United States; Certified Humane, created by Adele Douglas in 2003; and Global Animal Partnership (GAP), created in 2007 by the famous American chain supermarket, Whole Foods, among others. In Taiwan, several friendly livestock labels are used, for example, “Humane Monitoring”, launched in 2007; “Friendly Livestock”, created in 2012 by the Taiwan Society of Agricultural Standards; and “Friendly Production Certification” founded by the National Animal Industry Foundation to cooperate with traceable agricultural products and food safety certification. In 2018, the “Cage-free Alliance”, label launched by the Environment and Animal Society of Taiwan (EAST) and Carrefour to certify animal-friendly egg production, was extended in 2021 to the “Animal Welfare Label—EAST Certified” to cover livestock animals. The first dairy farm to apply and subsequently be approved through an audit was “JJ Farm” in Ruisui Township, Hualien County of Taiwan. Its approved product is “transparent fresh milk”, which is the first certified “fresh milk with an animal welfare label” in Taiwan. Transparent and clear information, such as the three main indicators of food safety, food quality, and trust, are provided through certification labels [4,5], reassuring consumers and improving their trust in certification labels, thereby increasing their consumption intention [6].

Grunert et al. [7] indicated that food safety is a factor for consumers to consider when buying organic pork, mainly because they believe that it has fewer residuals (pesticides, hormones, antibiotics) and is safer and healthier [8]. Moreover, Kehlbacher et al. [9] suggested that the price that consumers are willing to pay is affected by certification labels. Therefore, they included food safety and certification labels in the list of factors that affect consumer purchases. Most previous studies have focused on the impact of organic labels, nutrient tables, and food safety certifications on consumption behaviors [10,11,12,13,14,15,16,17]. This study discusses the individual demand level from the perspective of “animal welfare certification” labels, highlighting the importance of regular food certification for consumers, which is a different focus than previous studies that only emphasized the impact of organic labels, nutrient tables, and food safety certifications on consumption behaviors.

Previous studies have applied the theory of planned behavior (TPB) to various health behaviors (e.g., appropriate physical exercise and eating vegetables, fruits, and organic food) and to the purchase intention or decision concerning food certification (such as food safety and Halal certifications) [18,19,20,21,22]. However, questions about the topic of healthy food certification must still be clarified regarding the relationships among TPB components, such as whether consumers’ evaluations of health benefit certification will affect their attitudes toward it or whether a consumer’s behavior concerning this certification will affect their purchase intention. Studies have indicated that additional variables can be added to the TPB model [23,24] to increase the explanatory capacity of the model and determine the factors with relatively significant influences on behavioral intention.

This study aimed to identify the factors that influence individuals’ behavioral intention to purchase animal welfare-friendly products, using a framework of behavioral theory and empirical research. A more complete integrated model was proposed using the TPB as the basic theoretical framework and adding moral affection, health consciousness, and trust in labels to the main variables of attitudes, subjective norms, perceived behavioral control. This study will help clarify the consumption behavioral pattern of animal welfare-friendly products, which was mentioned but was not tested by previous studies, thereby filling this literature gap. Recommendations are made for the food industry, product evaluation units, marketing strategies, or business management that are expected to bring a safer and healthier dietary environment to consumers.

## 2. Literature Review and Research Hypotheses

### 2.1. TPB

TPB was proposed by Ajzen [25], who believes that attitudes, subjective norms, and perceived behavioral control jointly determine an individual’s behavioral intention. When an individual has positive attitudes toward a certain behavior, they have more supportive subjective norms of engaging in that behavior and stronger perceived behavioral control over that behavior in addition to higher intention to engage in that behavior. Jang et al. [26] found that attitudes, subjective norms, and perceived behavioral control positive influence the behavioral intention to visit an environmentally friendly restaurant. Al-Swidi et al. [18] discovered that subjective norms would directly influence the intention to buy organic food, interfere with the impact of attitudes and purchase intention, and simultaneously adjust the impact of perceived behavioral control and purchase intention.

### 2.2. Moral Affection

In recent years, the believers of moral consumerism have gradually focused on the topic of moral affection, suggesting that individual health behaviors are often affected by behavioral attitudes, such as labels, moral affection, consciousness, or beliefs [20,27,28]. Moral affection is defined as consumers’ inner emotional perception caused by product information with moral values (e.g., promoting human health, market order, and sustainable consumption) [29]. Moral affection is different from basic affection. Moral affection has complexity, links individual benefits with social welfare, and unconsciously generates moral behaviors, striking a fine balance between hedonistic and altruistic behaviors [30,31]. Therefore, when choosing healthy food related to personal benefits, the evaluation with moral affection can be induced through officially certified information [17,31], enabling the ability of moral affection to generate the appropriate intentional outcome [32,33].

Arvola et al. [20] applied TPB to a model that integrates emotional and moral attitudes to predict the purchase intention of organic food. Therefore, this study proposes the following hypotheses.

**H1a.**  *Moral affection significantly and positively influences attitudes.*

**H1b.**  *Moral affection significantly and positively influences subjective norms.*

**H1c.**  *Moral affection significantly and positively influences perceived behavioral control.*

According to the research results of this literature, this study assumes that consumers’ behavioral intention to purchase fresh milk with an animal welfare label is affected by three factors, namely, attitudes, subjective norms, and perceived behavioral control. Therefore, this study develops the following hypotheses.

**H2.**  *Attitudes significantly and positively influences the behavioral intention to purchase fresh milk with an animal welfare label.*

**H3.**  *Subjective norms significantly and positively influences the behavioral intention to purchase fresh milk with an animal welfare label.*

**H4.**  *Perceived behavioral control significantly and positively influences the behavioral intention to purchase fresh milk with an animal welfare label.*

### 2.3. Trust in Certification

Trust is a psychological state, referring to an individual’s inner perception that promotes interactions to compensate for the lack of information in risky situations or a lack of understanding of each other, thus enabling progress [34]. Garbarino and Johnson [35] mentioned that trust is the confidence of consumers in the reliability of companies’ product or service quality that is particularly evident in an uncertain consumption environment [36]. Trust plays an important role in the decision-making process of purchasing food [37] because merely a few consumers know the background of food production, while most cannot verify it [38]. Therefore, consumers need to believe that the food they purchase is authentic and genuine [39]. Singh and Sirdeshmukh [40] revealed that consumers’ evaluation of trust in an enterprise positively affects their loyalty.

Studies showed that consumers choose certified products mainly because they are believed to be healthier, more delicious [41,42], and more worthy of trust [11,43,44,45]. Certification label can not only convey trustworthy product information but also be an effective strategy to curb fraud of inauthentic products and help honest firms improve their income [46,47]. Consumers will increase their trust and purchase intention toward products with government certifications [13,48]. Chen [49] further verified that trust in suppliers (including manufacturers and retailers) is directly and positively correlated with food safety perception.

In response to the public attention on animal welfare, environmental sustainability, and food production ethics, developing friendly rearing has become a topic of international concern in past years. Consumers’ purchase intention and choices is significantly influenced by whether livestock products come from a farm that treats animals well. In this regard, the “animal welfare certification” label can be viewed as a reference for consumers to identify and purchase products. The “animal welfare certification” label uses strict standards developed by a fair third party. A scrupulous audit is performed on the basis of the standards to assist producers in improving a farm’s overall animal welfare and ensuring that the animals receive better treatment throughout their life cycles. By supporting livestock products labeled with an “animal welfare certification”, consumers can help ensure that producers and sellers pay attention to animal welfare, jointly improve situations for animals, and safeguard food safety and health. The animal welfare label system provides relevant information that consumers care about, such as animal welfare, environmental sustainability, and food safety. Although food safety problems have continued to emerge in recent years, the animal welfare label system plays an important role in improving consumers’ trust. Therefore, this study proposes the following hypotheses.

**H5a.**  *Trust in certification has a moderating effect on the relationship between attitudes and the behavioral intention to purchase fresh milk with an animal welfare label.*

**H5b.**  *Trust in certification has a moderating effect on the relationship between subjective norms and the behavioral intention to purchase fresh milk with an animal welfare label.*

**H5c.**  *Trust in certification has a moderating effect on the relationship between perceived behavioral control and the behavioral intention to purchase fresh milk with an animal welfare label.*

### 2.4. Health Consciousness

Hong [50] defined health consciousness as an individual’s psychological opinion of health and the intensity of perception rather than specific actions. Individuals who are health conscious will care about their health, actively improve or maintain their health and life quality, and engage in healthy behaviors to prevent illness [51].

Research has indicated that consumers will carry out judgment influenced by health consciousness in terms of product characteristics, such as functions, chemical processing, and planting method, and the results showed that respondents prefer functional and natural food [28,52]. Makatouni [53] pointed out that the most important motive for consumers to purchase organic food is that they value their health. Consumers’ health consciousness has a significant influential correlation with their attitudes and purchase intention toward organic food [54,55]. Therefore, this study proposes the following hypotheses.

**H6a.**  *Health consciousness has a moderating effect on the relationship between attitudes and the behavioral intention to purchase fresh milk with an animal welfare label*.

**H6b.**  *Health consciousness has a moderating effect on the relationship between subjective norms and the behavioral intention to purchase fresh milk with an animal welfare label.*

**H6c.**  *Health consciousness has a moderating effect on the relationship between perceived behavioral control and the behavioral intention to purchase fresh milk with an animal welfare label.*

## 3. Materials and Methods

### 3.1. Research Framework

**Figure 1:**
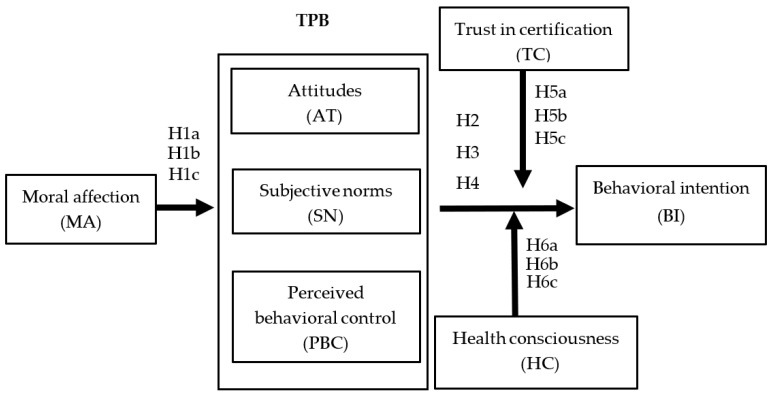
Conceptual framework and hypotheses of the study.

Considering this discussion of the literature, this study extends the framework of TPB by adding variables such as moral affection, trust in certification, and health consciousness in addition to the original TPB variables, namely, attitudes, subjective norms, perceived behavioral control, and behavioral intention. The framework is illustrated in Figure 1.

### 3.2. Research Questionnaire Design

The research questionnaire is divided into seven parts. Part One is moral affection, in reference to Sudbury-Riley and Kohlbacher [56], and has four questions. Part Two is the attitude scale, in reference to Wang et al. [57], and has three questions. Part Three is subjective norms, in reference to Han et al. [58], and has three questions. Part Four is perceived behavioral control, in reference to Awuni and Du [59] and Han et al. [58], and has five questions. Part Five is trust in certification, has a scale adapted from Mayer and Davis [60], and has six questions. Part Six is health consciousness, in reference to Gould [61] and Michaelidou and Hassan [52], and has five questions. Part Seven is behavioral intention, in reference to Chen [62] and Han et al. [58], and has four questions. Part Seven is respondents’ basic information, including gender, age, educational level, and average personal monthly income. All questions are measured using a 7-point Likert scale, except for the demographic variables. Respondents filled out the questionnaire on the basis of their personal perceptions or actual situations, with 1 indicating “strongly disagree” or the weakest feeling and 7 indicating “strongly agree” or the strongest feeling.

### 3.3. Sample and Data Collection

Given the progress over time and the popularization and convenience of the Internet, many social science researchers have shifted from paper-based distribution to collecting research data through the Internet and social media channels. Dommeyer and Moriarty [63] have indicated that utilizing the Internet to fill out questionnaires has advantages over the traditional random sampling survey method, such as lower cost, wider reach, and faster recovery time. This study used the SurveyCake platform to distribute the questionnaires over the Internet. Before the formal distribution of the questionnaire, a pre-test questionnaire was administered to enhance the reliability of the questionnaire by the respondents’ responses; 87 valid samples were collected in the pre-test. This study uses a reliability measurement on the research constructs to test whether the results are stable and consistent. The formal questionnaire survey was administered from 16 March to 15 April 2022. Researchers promoted and distributed the questionnaire through word-of-mouth, related websites, and personal Facebook and Line groups. In this study, the research subjects were consumers who consumed and purchased fresh milk with an animal welfare label. Currently, the following fresh milk products have acquired animal welfare labels: “Transparent Fresh Milk” of JJ Farm, “Hsu’s Fresh Milk” of Hsu’s Ranch, “Lucky Fresh Milk” of Lucky Ranch, and “Home Love Fresh Milk” of Home Love Ranch. Given the consideration of conforming to research ethics, on the first page of the questionnaire, the respondents were clearly informed about the research purpose and their ability to participate anonymously, so that they could respond to the questions free of privacy concerns.

A total of 741 questionnaires were recovered. After removing invalid samples with no variance among the questionnaire items or repeated answers, 653 valid samples remained. Respondents’ demographic statistics showed 459 women (70.3%) and 194 men (29.7%), and the sample distribution was in line with the fact that women are the main purchasers in most households [64,65]. Respondents were in the following age groups: 40–49 years was the largest, with 220 people (33.7%), followed by 197 people in the 30–39 years age group (30.2%), 122 people in the 50–59 years age group (18.7%), 65 people in the 18–29 years age group (9.9%), and 49 people aged 60 or older, who accounted for the smallest proportion (7.5%). In terms of marital status, 441 were married (67.5%) and 212 were unmarried (32.5%). In terms of education level, university (junior college) was the largest proportion with 371 people (56.8%), followed by high (vocational) school with 169 people (25.9%), research institute and higher with 72 people (11%), and middle school or lower with 41 people (6.3%). In terms of average personal monthly income, 368 people with NTD 45,001–65,000 accounted for the largest proportion (56.4%), followed by 127 people with NTD 25,001–45,000 (19.4%), 104 people with more than NTD 65,001 (15.9%), and 54 people with less than NTD 25,000 (8.3%). Regarding monthly purchases of fresh milk with an animal welfare label, 307 people made three to five purchases, accounting for the largest proportion (47.0%), followed by 225 people (34.5%) making two or fewer purchases, and 121 people (18.5%) making six or more purchases, representing the smallest proportion (18.5%).

From the perspective of demographic variables, the main customer profile for fresh milk with an animal welfare label was as follows: women, aged between 30–49 years, with a university (junior college) education, and with an average personal monthly income of NTD 45,001–65,000.

### 3.4. Methods of Data Analysis

This study adopted the quantitative research method, acquired data through a questionnaire survey, and used IBM SPSS Statistics 25.0 and AMOS v.24.0 software suites to perform the data analysis. In this study, the statistical analysis methods adopted include descriptive statistics (distribution table of number of times, percentage, mean, and standard deviation), reliability analysis, validity analysis, and structural equation modeling (SEM) to analyze the cause–effect relationship between the models of the hypotheses and the fitness of the overall model and to test the research hypotheses proposed by this study.

## 4. Analysis and Results

### 4.1. Test Results of Measurement Model Evaluation

This study has the following seven secondary variables: moral affection, attitudes, subjective norms, perceived behavioral control, health consciousness, trust in certification, and behavioral intention. First, confirmatory factor analysis was performed on each variable. According to the results, we removed the question items with a factor loading value less than 0.4. We then repeatedly performed confirmatory factor analysis to examine the RMSEA of the secondary variables; if the RMSEA was higher than 0.08, then the variable did not fulfill the fitness standard. We repeatedly modified the model on the basis of the question removal principle of the modifying indicator (MI) until the RMSEA of the secondary variable was less than 0.08 or became a saturated model. This question removal procedure was performed for each variable one by one. The six questions of “moral affection” were reduced to four, the three questions of “attitudes” remained the same, the four questions of “subjective norms” were reduced to three, the six questions of “perceived behavioral control” were reduced to five, the eight questions of “trust in certification” were reduced to six, the seven questions of “health consciousness” were reduced to five, and the five questions of “behavioral intention” were reduced to four, resulting in the final questionnaire of 30 question items.

After confirming the question items for the scale of the secondary variables, we performed a convergence validity test on each variable of the scale. Composite reliability (CR) is the combination of the reliability of all measurement variables and ranges from 0 to 1, with a higher value indicating a “higher proportion of actual variance in total variance”, that is, higher internal consistency. Fornell and Larcker [66] suggested that the CR value of a latent variable should be higher than 0.60. Average variance extracted (AVE) is most representative of the convergent validity of the latent variable. Fornell and Larcker [66] and Bagozzi and Yi [67] have suggested that the AVE value of the latent variable should be higher than 0.50.

In this study, the CR values of all variables of the scale were between 0.853 and 0.923, indicating good internal consistency within the scale. The AVE values were between 0.661 and 0.793, all exceeding the suggested value of 0.50 and indicating good convergent validity of the scale. The standardized regression weighted coefficients of all question items were between 0.765 and 0.927, with a t value higher than 1.96, indicating significance. Table 1 lists the factor loading CR and AVE values of the variables. According to the table content, all variables of this questionnaire fulfilled the requirement for convergent validity; therefore, the measurement model has good internal quality.

Finally, we tested the discriminant validity of the variables. According to the suggestions by Hair et al. [68], the correlation coefficient between two different concepts should be smaller than the square root of the AVE value of each concept. Table 2 provides a comparison of the correlation coefficient of all variables in this study and the square root of the variables’ AVE values. These square root values are larger than the correlation coefficient between two variables, meeting the suggested standard by Hair et al. [68]. This finding shows that discriminant validity exists among the variables of this study. The test result of this study’s measurement model evaluation indicates that this model has good internal and external quality.

### 4.2. Structural Model Analysis

This study conducted a structural model to test the hypothetical relationship of the proposed model using the maximum likelihood method. The model fit index determines whether the sample data conforms to the suggested structural equation model. The structural model provided a good fit to the data after using Bollen–Stine bootstrap model fit: χ^2^/*df* = 1.285, RMSEA = 0.043, CFI = 0.971, TLI = 0.985, GFI = 0.957. All of the evaluation indicators meet the criteria. Therefore, the overall model of this study has good fitness.

When using SEM to analyze the indirect effect, the bootstrap method is the important way to obtain the confidence interval of it. Indirect effects were tested using bootstrapped confidence intervals (CI) using 1000 resamples [69]. If CIs do not contain 0, indirect relationships are significant, indicating significant mediating effect [70]. The results are presented in Table 3. Moral affection significantly and positively influences attitudes (H1a: β = 0.906, CI = [0.871, 0.936]), subjective norms (H1b: β = 0.851, CI = [0.776, 0.894]), and behavioral control (H1c: β = 0.624, CI = [0.547, 0.715]). Attitudes significantly and positively influences behavioral intention (H2: β = 0.521, CI = [0.429, 0.648]). Subjective norms significantly and positively influences behavioral intention (H3: β = 0.306, CI = [0.186, 0.421]). Finally, Perceived behavioral control significantly and positively influences behavioral intention (H4: β = 0.137, CI = [0.062, 0.237]). Overall, the structural model analysis results show that all hypotheses are supported.

### 4.3. Moderating Effect of Trust in Certification and Health Consciousness

The moderating effects were determined by calculating the mean-centering indicator values before the moderator variable multiplication with the predictor variables [71]. As shown in Table 4, most hypotheses are not supported. We use trust in certification as the moderating variable; attitudes, subjective norms, and perceived behavioral control of TPB as independent variables; and behavioral intention as the dependent variable to verify the following hypotheses. Trust in certification has a moderating effect on the relationship between attitudes and behavioral intention (H5a: β = −0.025, *p* = 0.113); thus, the hypothesis is not supported. Trust in certification has a moderating effect on the relationship between subjective norms and behavioral intention (H5b: β = 0.037, *p* = 0.012), indicating that a moderating effect exists or that a one unit increase in trust in certification results in the slope of the influence of subjective norms on behavioral intention increasing by 0.037 unit; thus, the hypothesis is supported. Trust in certification has a moderating effect on the relationship between perceived behavioral control and behavioral intention (H5c: β = 0.028, *p* = 0.001), indicating that the moderating effect exists or that a one unit increase in trust in certification results in the slope of the influence of perceived behavioral control on behavioral intention increasing by 0.028 unit. Furthermore, we use health consciousness as a moderating variable; attitudes, subjective norms, and perceived behavioral control of TPB as independent variables; and behavioral intention as the dependent variable to verify the following hypotheses. Health consciousness has a moderating effect on the relationship between attitudes and behavioral intention (H6a: β = 0.002, *p* = 0.871); thus, the hypothesis is not supported. Health consciousness has a moderating effect on the relationship between subjective norms and behavioral intention (H6b: β = −0.054, *p* = 0.002); thus, the hypothesis is not supported. Health consciousness has a moderating effect on the relationship between perceived behavioral control and behavioral intention (H6c: β = −0.048, *p* = 0.004); thus, the hypothesis is not supported.

The following results are concluded according to the analyses: moral affection significantly and positively influences attitudes, subjective norms, and perceived behavioral control; moreover, attitudes, subjective norms, and perceived behavioral control significantly and positively influence behavioral intention. When using trust in certification and health consciousness as moderating variables, it is found that trust in certification has a moderating effect on subjective norms, behavioral intention, and perceived behavioral control, and the influences of the other variables are nonsignificant. Therefore, Hypotheses H5a, H6a, H6b, and H6c are not supported.

## 5. Discussion

This study adopts ETPB to explore consumers’ behavioral intentions to purchase fresh milk with an animal welfare label. According to the research findings, attitudes, subjective norms, and perceived behavioral control have significant and positive influences on the behavioral intention to purchase fresh milk with an animal welfare label. This finding is in line with that of most studies adopting TPB [26,72,73,74,75,76]. Ajzen [19] found that consumers’ behavior of choosing healthy food is the intention on the basis of attitudes, subjective norms, and perceived behavioral control—factors that depend on the belief of behavior, norms, and control. Their study results indicated that people’s behavioral intention to engage in a certain behavior is influenced by all or part of the factors, such as attitudes, subjective norms, and perceived behavioral control.

Moreover, consumers’ moral affection for fresh milk with an animal welfare label positively influences their behavioral intention to purchase fresh milk with an animal welfare label through attitudes, subjective norms, and perceived behavioral control. In other words, the following factors positively influence consumers’ behavioral intention to purchase fresh milk with an animal welfare label: consumers perceive the moral affection for animal welfare-friendly products and hold a positive attitudes toward fresh milk with an animal welfare label; their families and friends have positive opinions and comments on fresh milk with an animal welfare label; and they believe they have sufficient money, time, and information about animal welfare-friendly products. Previous literature linked moral affection to the ecological label, social responsibility, and the traceable label, believing that doing so affects consumers’ attitudes and expectations [77,78,79]. Regarding the production of farm animals, many consumers hope that the meat production process can consider animal welfare and social morality properties [7]. In recent years, studies started to discuss the relationship between moral affection and related animal-friendly attitudes and the selection of animal welfare-friendly products [80,81,82,83,84,85]. Consumers pay more attention to moral values, such as product origin and actual benefits, and use this moral standard as a motivation for purchase decisions [32]. The research findings show that moral affection valuing social responsibility improves people’s behavioral intention toward animal welfare-friendly products. That is to say, products with an animal welfare certification label, which focus on humane rearing and green consumption consciousness and feature freshness and food safety guarantees (no drug testing), improve people’s behavioral intention toward animal welfare-friendly products. In particular, women who have a higher education and a decent income are more interested and supportive.

Furthermore, this study extends the variables of trust in certification and health consciousness. According to this study’s findings, fresh milk with an animal welfare label has trust in certification because of its friendliness toward the environment and animals, food quality, and safety, which in turn positively influence consumers’ behavioral intention to purchase fresh milk with an animal welfare label. This result is also in line with the call for strengthening trust in certification given the moral perception and positive attitudes toward fresh milk with an animal welfare label, probably because consumers can quickly and clearly identify the certification label [10,86]; moreover, it is consistent with previous literature regarding the focus on examining product labels [15,87].

Studies have further shown that consumers tend to have more trust in a green product when its green product certification label is accredited by a third party (especially a public institution) [88]. However, consumers consider an increasing number of trust properties in their purchase decisions, and these properties cannot be deduced directly through search or experience, such as safety, nutrition, environmental protection, and animal welfare. Traditionally, branding, marketing, and advertising were used to convey such reputation properties of certain foods, affecting consumers’ choices [89,90,91]. Consumption of livestock products differentiated by production and processing methods generally depends on consumer trust, which is influenced by the consumer’s level of knowledge, information, and education [92,93,94]. When consumers purchase fresh milk with an animal welfare label, they still care most about product quality, sanitary standards, and traceability, as well as the trust generated by the producer’s reputation. Further, consumers also gradually pay attention to the humane rearing and growing environment of pigs. Dairy farms with an honest image are more trusted and recognized by general consumers.

Finally, this study finds that consumers’ perception of animal welfare certification labels is triggered by moral affection, and they then form a positive attitudes toward certification labels, strengthening the health consciousness of fresh milk with an animal welfare label and, thereby, achieving the final behavioral intention. This result is consistent with that of other researchers’, indicating that the external characteristics of health benefits can indeed trigger consumers’ perceived interest [95,96]. Consumers are more health conscious toward functional or 100% natural foods (e.g., certified healthy foods, whole grains, and vegetables) [52,97,98]. Studies have highlighted that health-conscious and -oriented consumers and those with greater knowledge of nutrition usually read instructions on food labels [99,100]. Consumers who focus on health are more willing to buy organic food [54,55].

In particular, when a benefit is verified, consumers’ inner moral affection will continuously brew and perceive the benefits of healthy food, promoting their attitudes, consciousness, and behavioral intentions. Therefore, animal welfare certification labels can be regarded as powerful inspirational clues [101]. These findings will be informative for dairy product manufacturers’ marketing strategies.

## 6. Conclusions

### 6.1. Conclusion of This Study

According to these findings, when purchasing fresh milk with an animal welfare label, consumers focus more on product quality, sanitary standards, and traceability, as well as producer credibility, among others, representing the trust generated by fresh milk with an animal welfare label. Because this key factor influences the consumption intention of animal welfare-friendly products, relevant government authorities should strictly supervise the audit of animal welfare certification labels to maintain the public’s trust.

Moreover, targeted marketing strategies can be developed for high-income groups, such as emphasizing that dairy products conform to friendly rearing, environmental sustainability, and other consumer ethics concepts, and increasing prices to ensure that consumers know that their willingness to pay is higher. In doing so, dairy farms and dairy product manufacturers will also have higher revenues, enabling them to be more able to improve the aspects of environmental protection and friendly rearing, among others, thus forming a benign cycle of sustainable agricultural development and establishing a mutually beneficial coexistence model of businesses, consumers, and environment.

### 6.2. Limitations and Future Directions

An important limitation of this study is the perception of label information. Although this study has clearly explained the definition of the “animal welfare certification” label at the beginning of the questionnaire, this topic is new, and not every respondent shares the same understanding. The questionnaire design does not investigate respondents’ identification and understanding of the “animal welfare certification” label, which may make some respondents to have biases when answering the questions. Therefore, we recommend that future studies first test respondents’ knowledge level of labels.

Second, this study takes TPB as its core and incorporates variables, such as moral affection, health consciousness, and trust in certification, to discuss Taiwanese people’s thoughts on purchasing animal welfare-friendly products and the factors influencing their purchase decisions. Future studies can incorporate variables, such as perceived quality, perceived risk, and perceived interest, to make the overall research more complete. Additionally, we recommend that future studies adopt contingent valuation method or choice experiment method among other methods to ensure more precise research and discussions on consumers’ willingness to pay for fresh milk with an animal welfare label, thus filling the gap in research data on the willingness to pay for animal welfare-friendly products in Taiwan.

## Figures and Tables

**Table 1:** Summary of reliability test on measurement model.

Variable/Question Item	Mean	SD	Standardized Factor Loading	AVE	CR
Moral affection (MA)	5.503	0.785		0.661	0.895
1. I believe that dairy farms should not illegally use banned substances (including antibiotics and clenbuterol), which is a necessary rule to ensure human and animal health	5.531	0.702	0.892 ***		
2. I believe that it socially responsible for dairy farms to move toward low-polluting development (e.g., manure compost)	5.083	0.695	0.841 ***		
3. I believe that it is necessary for dairy farms to comply with animal welfare laws	5.754	0.833	0.895 ***		
4. I believe that purchasing fresh milk with an animal welfare label produced by an environmentally friendly dairy farm is a responsible act for environmental protection	5.642	0.912	0.906 ***		
Attitudes (AT)	5.944	0.726		0.759	0.853
1. I believe it is a good idea to purchase fresh milk with an animal welfare label	5.642	0.782	0.792 ***		
2. I believe it is a wise choice to purchase fresh milk with an animal welfare label	5.917	0.703	0.815 ***		
3. I like the idea of purchasing fresh milk with an animal welfare label	6.272	0.693	0.904 ***		
Subject norms (SN)	5.271	0.533		0.708	0.907
1. Most people important to me think I should purchase fresh milk with an animal welfare label	5.828	0.641	0.892 ***		
2. Most people I value are more willing to purchase fresh milk with an animal welfare label	4.934	0.783	0.806 ***		
3. The degree of influence from individuals or groups will significantly affect my purchase of fresh milk with an animal welfare label	5.052	0.175	0.896 ***		
Perceived behavioral control (PBC)	5.562	0.804		0.705	0.901
1. I am willing to pay more to purchase fresh milk with an animal welfare label for the sake of the ecosystem	5.754	0.682	0.906 ***		
2. I believe that I can purchase fresh milk with an animal welfare label for ecological reasons	5.621	0.761	0.886 ***		
3. I believe I have full confidence in the credibility of fresh milk with an animal welfare label	5.581	1.052	0.895 ***		
4. I believe it is the right choice to purchase fresh milk with an animal welfare label	5.762	0.842	0.902 ***		
5. I can decide independently whether to choose fresh milk with an animal welfare label	5.092	0.681	0.874 ***		
Trust in certification (TC)	5.861	0.706		0.793	0.871
1. I think the quality of fresh milk with an animal welfare label is better guaranteed	5.913	0.748	0.891 ***		
2. I think the traceability of fresh milk with an animal welfare label can find the accountable unit for substandard fresh milk	6.012	0.693	0.927 ***		
3. I think if a dairy farm has fresh milk with an animal welfare label, it means it is committed to continuously improving its business and production	5.921	0.726	0.925 ***		
4. I think the fresh milk produced by dairy farms adopting humane management is more reassuring	5.865	0.748	0.894 ***		
5. I think it is humane to alleviate the pain of animals as much as possible during the slaughtering process	5.761	0.695	0.889 ***		
6. I think a dairy farm that puts effort into refining its fresh milk is a trustworthy producer	5.694	0.681	0.817 ***		
Health consciousness (HC)	5.154	0.422		0.684	0.923
1. I often reflect on my health status	4.583	0.398	0.801 ***		
2. I am very conscious about my health	5.012	0.401	0.812 ***		
3. I am vigilant of changes in my health	5.137	0.382	0.765 ***		
4. I usually know my health status	5.493	0.435	0.858 ***		
5. I am responsible for my health status	5.547	0.496	0.906 ***		
Behavioral intention (BI)	5.827	0.930		0.764	0.908
1. I will prioritize the consideration to purchase fresh milk with an animal welfare label	5.798	0.854	0.887 ***		
2. I will still purchase fresh milk with an animal welfare label if I can choose again	5.972	1.022	0.892 ***		
3. I will recommend to my friends and families to purchase fresh milk with an animal welfare label	5.860	0.858	0.874 ***		
4. I will still purchase fresh milk with an animal welfare label even though it is more expensive	5.679	0.986	0.885 ***		

Note: *** *p* < 0.001.

**Table 2:** Correlation coefficient and AVE square root of measurement model.

	1	2	3	4	5	6	7
1. MA	0.813						
2. AT	0.702	0.871					
3. SN	0.716	0.803	0.841				
4. PBC	0.626	0.518	0.791	0.840			
5. TC	0.592	0.206	0.206	0.525	0.891		
6. HC	0.605	0.757	0.795	0.548	0.018	0.827	
7. BI	0.704	0.768	0.801	0.647	0.494	0.653	0.874

Note 1: Figures on the diagonal line are the AVE square roots of the latent variables. Note 2: MA = Moral affection, AT = Attitudes, SN = Subject norms, PBC = Perceived behavioral control, TC = Trust in certification, HC = Health consciousness, BI = Behavioral intention.

**Table 3:** Results of the path analysis and hypothesis testing.

Hypothesized Paths	Non-Standardized Coefficient	S.E.	*p*	Standardized Coefficient	95% CI		Explanatory Capacity (R^2^)	Test Results
					Lower Bound	Upper Bound		
H1a: MA→AT	0.876	0.057	0.001	0.906	0.871	0.936	0.836	YES
H1b: MA→SN	0.916	0.062	0.001	0.851	0.776	0.894	0.708	YES
H1c: MA→PBC	0.625	0.071	0.002	0.624	0.547	0.715	0.412	YES
H2: AT→BI	0.601	0.048	0.002	0.521	0.429	0.648	0.692	YES
H3: SN→BI	0.342	0.042	0.001	0.306	0.186	0.421	0.783	YES
H4: PBC→BI	0.176	0.033	0.002	0.137	0.062	0.237	0.708	YES

**Table 4:** Moderating effect.

Hypothesis	MOD	IV	DV	Non-Standardized Coefficient	S.E.	Z-Value	*p*	Test Results
H5a	Trust in certification	AT	BI	−0.025	0.014	1.42	0.113	NO
H5b		SN		0.037	0.013	2.18	0.012	YES
H5c		PBC		0.028	0.012	3.07	0.001	YES
H6a	Health consciousness	AT		0.002	0.024	1.06	0.871	NO
H6b		SN		−0.054	0.019	2.14	0.002	NO
H6c		PBC		−0.048	0.018	2.62	0.004	NO

## Data Availability

The data that support the findings of this study are available from the corresponding author, H.-S.C., upon reasonable request.
